# Proper position of single and large (≥5 cm) hepatocellular carcinoma in Barcelona Clinic Liver Cancer classification

**DOI:** 10.1097/MD.0000000000034639

**Published:** 2023-10-13

**Authors:** Hyo-Sin Kim, Soo Jin Na Choi, Ho Kyun Lee

**Affiliations:** a Department of Surgery, Chonnam National University Medical School and Hospital, Gwangju, Republic of Korea.

**Keywords:** BCLC, hepatocellular carcinoma, propensity score-matched groups, survival outcome

## Abstract

The purpose of this study was to evaluate the proper position of single large hepatocellular carcinoma (HCC) in the Barcelona Clinic Liver Cancer (BCLC) staging system. The data were collected from the nationwide multicentre database of the Korean Liver Cancer Association. Patients with single large (≥5 cm) HCC were separated from BCLC stage A patients and designated as Group X. The remaining BCLC stage A and stage B patients were classified as Group A and Group B, respectively. The survival outcomes of propensity score-matched groups were compared. Among the 3965 randomly selected patients, the number of patients in Group X, Group A, and Group B was 414, 2787, and 760, respectively. TriMatch analysis allowed us to obtain 116 well-balanced triplets. The 1-, 3-, and 5-year overall survival rates in Group X were worse than in Group A (91%, 71%, and 48% vs 90%, 78%, and 64%, respectively; *P* < .000). However, the rates were not different compared with those in Group B (91%, 71%, and 48% vs 90%, 69%, and 48%, respectively; *P* < .09). In multivariate analysis, Group X, Group B, age over 60 years, prothrombin time-international normalized ratio, and creatinine level were independent predictors of worse overall survival. Our findings suggest that Group X should be relocated to BCLC stage B rather than BCLC stage A.

## 1. Introduction

Hepatocellular carcinoma (HCC) is a leading cause of cancer-related deaths with a growing global burden.^[1]^ Effective management of HCC is essential for improving patient outcomes, and the Barcelona Clinic Liver Cancer (BCLC) staging system is a widely used tool for determining the appropriate treatment strategy for HCC patients.^[2]^ The BCLC system categorizes HCC patients into 5 stages (0, A, B, C, and D) based on tumor characteristics, liver function, and performance.^[2–4]^

One critical aspect of HCC management is the accurate classification of tumors within the BCLC system as it can directly affect treatment decision and prognosis. Currently, single large (≥5 cm) HCC is classified as BCLC stage A.^[3]^ However, recent studies have questioned the appropriateness of this classification, suggesting that single large HCC might have different outcomes compared with those of other stage A tumors.^[5,6]^

The purpose of this study was to evaluate the proper position of single large HCC in the BCLC staging system. Specifically, we aimed to determine whether the survival outcomes of patients with single large (≥5 cm) HCC are more closely aligned with those of patients in BCLC stage A or B. A better understanding of the prognosis for single large HCC could help refine the BCLC staging system and improve treatment recommendations for these patients.

To achieve this, we analyzed data from the nationwide multicentre database of the Korean Liver Cancer Association. We compared the overall survival rates of propensity score-matched patients with single large (≥5 cm) HCC, reclassified as Group X, with those of the remaining patients in BCLC stages A and B.

In this study, we present the results of our study, discuss the clinical implications of our findings, and suggest potential revisions to the BCLC staging system based on our results.

## 2. Methods

### 2.1. Patients and methods

#### 1.2.1. Study design and population.

This study was a nationwide multicentre registry-based comparative analysis of patients with HCC categorized into 3 groups: Group X, single nodule > 5 cm in diameter; Group A, single nodule ≤ 5 cm or 2 or 3 nodules ≤ 3 cm in diameter; Group B, 2 or 3 nodules > 3 cm in diameter. The study was conducted following the ethical guidelines of the 1975 Declaration of Helsinki and was approved by the Institutional Review Board of Chonnam National University Hospital.

### 2.2. Registry and data collection

Data were obtained from the Korean Central Cancer Registry, a governmental organization with a statutory nationwide cancer registry. The population-based registry was established in 1999 and accounts for over 95% of all cancer cases. This study used a retrospective, randomly selected, nationwide database from the Korea Central Cancer Registry, focusing on newly diagnosed HCC cases from 2008 to 2014.

The database included information on age, sex, date of diagnosis, related etiology (hepatitis B or C virus (HBV or HCV), alcoholic liver disease, etc.), Child-Pugh classification, Model for End-stage Liver Disease (MELD) score, portal vein invasion, tumor number, tumor size (defined as the diameter of the largest lesion), and BCLC staging. Individuals with missing data for any of the above variables were excluded from the analysis.

### 2.3. Outcomes

The primary outcome was long-term overall survival. Analyses were performed on both unmatched and propensity score-matched groups. Prognostic factors were compared by univariate and multivariate analyses for all patients in the matched groups.

### 2.4. Statistical analysis

Propensity score matching (1:1:1) using TriMatch version 0.9.9 from R (The R Foundation for Statistical Computing, Vienna, Austria) was performed to control for imbalances between the 3 groups.^[7,8]^ Kurtosis and skewness were calculated for each continuous variable to distinguish parametric and non-parametric variables, which are presented as the mean and standard deviation and median and interquartile (25–75) range, respectively. ANOVA, Kruskal–Wallis, and χ^2^ tests were used to compare triplets for multiple independent factors as appropriate. Student’s unpaired *t* test, Mann–Whitney *U* test, and Pearson’s χ^2^ test were used to compare 2 independent factors. The 3 groups were compared overall and pairwise using pre- and intraoperative variables as well as the chosen outcomes to evaluate haemostasis control. Statistical significance was set as *P* ≤ .05. Analyses were performed with RStudio and SPSS version 20.0 (SPSS, Chicago, IL).

## 3. Results

### 3.1. Patient selection

**Figure 1.**
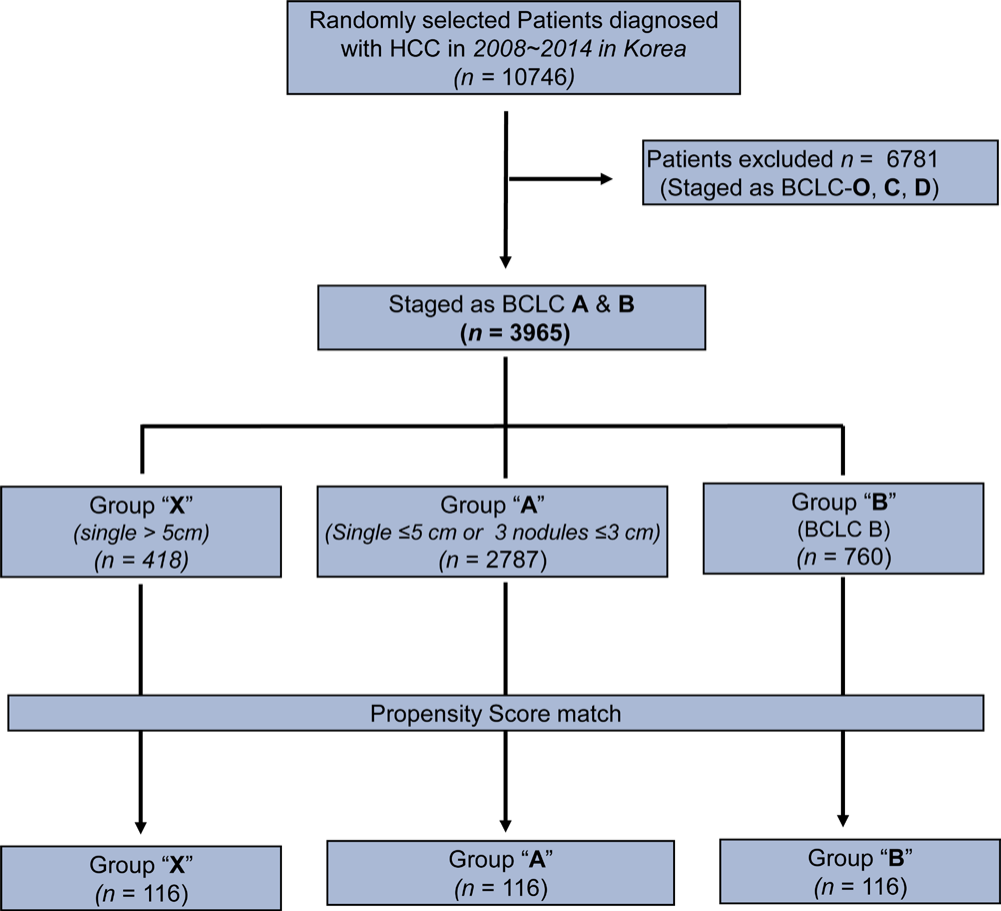
Flow chart of patient selection. BCLC = Barcelona Clinic Liver Cancer, HCC = hepatocellular carcinoma.

A total of 10,746 patients diagnosed with HCC in Korea between 2008 and 2014 were selected through systematic random sampling from the Korea Central Cancer Registry (Fig. 1). From this sample, 3965 patients were chosen, including 3205 with early-stage (BCLC-A) and 760 with intermediate-stage (BCLC-B) HCC. Group X comprised 418 patients with a single nodule > 5 cm in diameter, Group A consisted of 2787 patients with a single nodule ≤ 5 cm or 2 or 3 nodules ≤ 3 cm in diameter, and Group B included 760 patients with 2 or 3 nodules > 3 cm in diameter. After propensity score matching, each group contained 116 patients.

### 3.2. General characteristics

The average age was 60.29 ± 11.09 years, and 3055 (77.0%) patients were male (Table 1). In terms of liver disease etiology, 2417 (61.0%) patients had HBV-related disease, 543 (13.7%) patients had HCV-related disease, and 1177 (29.7%) patients had alcohol-related disease. Based on Child-Pugh classification, 3392 (85.5%) patients were classified as Class A, and 573 (14.5%) patients were classified as Class B. HCC treatment was divided into the curative (hepatectomy, radiofrequency ablation, and liver transplantation) and non-curative (transarterial chemoembolization, chemotherapy, radiation therapy, and no treatment) groups (Table 1). Among the treated patients, 1183 (29.8%) patients underwent hepatectomy, 575 (14.5%) patients received radiofrequency ablation, 44 (1.1%) patients underwent liver transplantation, 1862 (47.0%) patients underwent transarterial chemoembolization, 28 (0.7%) patients received chemotherapy, 11 (0.3%) patients received radiation therapy, and 262 (6.6%) patients did not receive any treatment.

**Table 1:** General characteristics (N = 3965).

Age, years (SD)	60.29 (11.09)
Sex, n (%)	
Male	3055 (77.0)
Female	910 (23.0)
Etiology, n (%)	
HBV-related	2417 (61.0)
HCV-related	543 (13.7)
Alcohol-related	1177 (29.7)
Child-Pugh classification, n (%)	
A	3392 (85.5)
B	573 (14.5)
MELD score, (SD)	8.89 (3.11)
Number of tumors, n (SD)	1.65 (1.17)
Max tumor size, cm (SD)	3.55 (2.38)
mBCLC, n (%)	
A	2787 (70.3)
B	760 (19.2)
X	418 (10.5)
Treatment, n (%)	
Hepatectomy	1183 (29.8)
RFA	575 (14.5)
Liver transplantation	44 (1.1)
TACE	1862 (47.0)
Chemotherapy	28 (0.7)
Radiation therapy	11 (0.3)
No treatment	262 (6.6)

HBV = hepatitis B virus, HCV = hepatitis C virus, MELD = Model for End-stage Liver Disease, RFA = radiofrequency ablation, TACE = transarterial chemoembolization.

### 3.3. Characteristics of 3 groups

Baseline characteristics before and after propensity score matching are summarized in Table 2. Significant differences in several baseline variables were observed among the 3 groups before matching, such as sex, age, etiology (HBV, HCV, and alcohol), Child-Pugh classification, preoperative laboratory findings (platelet count, albumin, bilirubin, and prothrombin time-international normalized ratio (PT-INR)), MELD score, and treatment approach (curative vs non-curative). However, no significant differences were observed among the 3 groups after matching.

**Table 2:** Baseline characteristics before and after propensity score matching.

	Before matching				After matching			
	X	A	B	*P* †	X	A	B	*P* †
	(N = 418)	(N = 2787)	(N = 760)		(N = 116)	(N = 116)	(N = 116)	
Age								
≥ 60 yr, n (%)	245 (58.6)	1409 (50.6)	403 (53.0)	.007	63 (54.3)	63 (54.3)	66 (56.9)	.901
<60 yr, n (%)	173 (41.4)	1378 (49.4)	357 (47.0)		53 (45.7)	53 (45.7)	50 (43.1)	
Sex				<.001				.892
Male, n (%)	345 (82.5)	2080 (74.6)	630 (82.9)		90 (77.6)	88 (75.9)	91 (78.4)	
Female, n (%)	73 (17.5)	707 (25.4)	130 (17.1)		26 (22.4)	28 (24.1)	25 (21.6)	
Number of tumors, n (SD)*	1 (0.00)	1.24 (0.51)	3.54 (1.35)	<.001‡	1 (0.00)	1.11 (0.31)	3.01 (1.28)	<.001‡
Max tumor size, cm (SD)*	7.57 (2.14)	2.53 (1.00)	5.09 (2.88)	<.001‡	6.83 (1.57)	2.18 (0.86)	4.47 (2.19)	<.001‡
Child-Pugh classification				<.001				.906
A, n (%)	390 (93.3)	2370 (85.0)	632 (83.2)		101 (87.1)	99 (85.3)	101 (87.1)	
B, n (%)	28 (6.7)	417 (15.0)	128 (16.8)		15 (12.9)	17 (14.7)	15 (12.9)	
HBV-related, n (%)	210 (50.2)	1748 (62.7)	459 (60.4)	<.001	63 (54.3)	57 (49.1)	67 (57.8)	.415
HCV-related, n (%)	36 (8.6)	397 (14.2)	110 (14.5)	.006	10 (8.6)	21 (18.1)	20 (17.2)	.078
Alcohol-related, n (%)	128 (30.6)	763 (27.4)	286 (37.6)	<.001	36 (31.0)	40 (34.5)	34 (29.3)	.689
Platelets (≤100,000 vs >10,000/µL)	46 (11.0)	946 (33.9)	212 (27.9)	<.001	34 (29.3)	36 (31.0)	33 (28.4)	.908
Albumin ≤ 3.5 g/dL, n (%)	83 (19.9)	736 (26.4)	231 (30.4)	<.001	42 (36.2)	42 (36.2)	39 (33.6)	.893
Bilirubin ≥ 1.2 mg/dL, n (%)	76 (18.2)	806 (28.9)	217 (28.6)	<.001	36 (31.0)	36 (31.0)	38 (32.8)	.948
PT-INR ≤ 1.2, n (%)	131 (31.3)	1338 (48.0)	369 (48.6)	<.001	64 (55.2)	60 (51.7)	61 (52.6)	.861
Creatinine ≥ 1.2 mg/dL, n (%)	61 (14.6)	316 (11.3)	80 (10.5)	.095	15 (12.9)	14 (12.1)	16 (13.8)	.926
MELD score ≥ 10	47 (11.2)	562 (20.2)	146 (19.2)	<.001	27 (23.3)	28 (24.1)	27 (23.3)	.984
Treatment				<.001				.986
Non-curative, n (%)	214 (51.2)	1323 (47.5)	626 (82.4)		81 (69.8)	82 (70.7)	81 (69.8)	
Curative, n (%)	204 (48.8)	1464 (52.5)	134 (17.6)		35 (30.2)	34 (29.3)	35 (30.2)	

HBV = hepatitis B virus, HCV = hepatitis C virus, MELD = Model for End-stage Liver Disease, PT-INR = prothrombin time-international normalized ratio.

* Diameter and number based on radiological findings.

† χ^2^ or Fisher’s exact test, except.

‡ ANOVA test.

### 3.4. Overall survival

**Figure 2.**
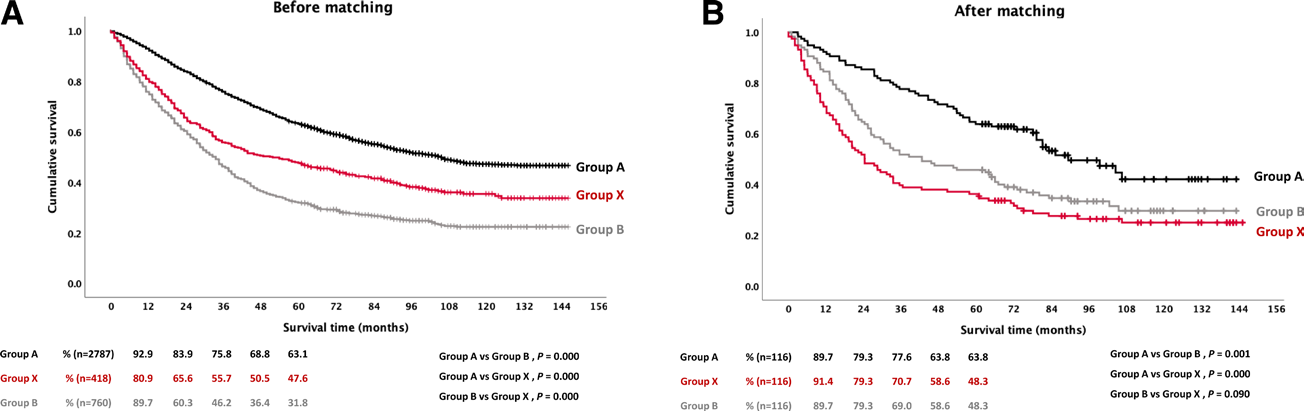
A. Overall survival rates of patients in 3 groups before propensity score matching. B. Overall survival rates of patients in 3 groups after propensity score matching.

Before matching, the 1-, 2-, 3-, and 5-year overall survival rates were the highest in Group A (92.9%, 83.9%, 75.8%, and 63.1%), followed by Group X (80.9%, 65.6%, 55.7%, and 47.6%) and Group B (89.7%, 60.3%, 46.2%, and 31.8%) (*P* < .001) (Fig. 2A). After matching, the 1-, 2-, 3-, and 5-year overall survival rates remained the highest in Group A (89.7%, 79.3%, 77.6%, and 63.8%) (*P* < .001). However, no significant difference was observed between Group X (91.4%, 79.3%, 70.7%, and 48.3%) and Group B (89.7%, 79.3%, 69.0%, and 48.3%) (*P* = .090) (Fig. 2B).

### 3.5. Analysis of prognostic factors

Prognostic factors were analyzed using a Cox proportional hazard model for variables obtained from the database Table 3).

**Table 3:** Cox proportional hazard analysis of prognostic factors for overall survival (N = 348).

	Univariate analysis		Multivariate analysis	
	Hazard ratio	*P*	Hazard ratio	*P*
Age (≥ 60 vs < 60 years)	0.63 (0.48–0.84)	.001	1.43 (1.06–1.92)	.019
Sex (M vs F)	1.03 (0.75–1.42)	.831		
Child-Pugh grade (B vs A)	0.47 (0.33–0.67)	<.001	1.34 (0.84–2.14)	.213
HBV-related	1.47 (1.12–1.92)	.004	0.84 (0.63–1.17)	.231
HCV-related	0.90 (0.62–1.30)	.594		
Alcoholic liver disease	0.77 (0.58–1.01)	.067		
Platelets (≤100,000 vs >100,000/µL)	0.62 (0.47–0.82)	.001	1.15 (0.84–1.57)	.357
Albumin (<3.5 vs ≥3.5 g/dL)	0.56 (0.43–0.74)	<.001	1.25 (0.89–1.74)	.184
Total bilirubin (≥1.2 vs <1.2 mg/dL)	0.65 (0.50–0.86)	.003	1.13 (0.78–1.62)	.513
PT-INR (≥1.2 vs <1.2)	0.54 (0.41–0.71)	<.001	1.38 (1.02–1.88)	.035
Creatinine (≥1.2 vs <1.2 mg/dL)	0.67 (0.46–0.98)	.042	1.61 (1.00–2.60)	.048
MELD score (≥10 vs <10)	0.55 (0.41–0.74)	<.001	1.09 (0.69–1.73)	.692
Group A				
Group B	1.73 (1.22–2.45)	.002	1.82 (1.28–2.60)	.001
Group X	2.25 (1.60–3.17)	<.001	2.61 (1.84–3.69)	<.001
Treatment (non-curative vs curative)	0.35 (0.24–0.49)	<.001	2.54 (1.74–3.72)	<.001

Values in parentheses are the 95% confidence intervals.

HBV = hepatitis B virus, HCV = hepatitis C virus, MELD = Model for End-stage Liver Disease, PT-INR = prothrombin time-international normalized ratio.

In univariate analysis, age over 60 years, Child-Pugh grade B, HBV-related liver disease, platelet count below 100,000/µL, albumin level below 3.5 g/dL, total bilirubin level over 1.2 mg/dL, PT-INR over 1.2, creatinine level above 1.2 mg/dL, MELD score over 10, Group B compared with Group A, Group X compared with Group A, and curative treatment were identified as significant prognostic factors.

In multivariate analysis, age over 60 years (HR 1.43, 1.06–1.92; *P* = .019), PT-INR over 1.2 (HR 1.38, 1.02–1.88; *P* = .035), creatinine level above 1.2 mg/dL (HR 1.61, 1.00–2.60; *P* = .048), Group B compared with Group A (HR 1.82, 1.28–2.60; *P* = .001), Group X compared with Group A (HR 2.61, 1.84–3.69; *P* < .001), and curative treatment (HR 2.54, 1.74–3.72; *P* < .001) remained as independent risk factors for worse overall survival.

## 4. Discussion

In our study, we found that the overall survival of patients with single large HCC (>5 cm) was significantly worse than that of Group A (originally BCLC-A) and similar to that Group B (originally BCLC-B). Multivariate analysis also revealed that single large HCC and Group B were risk factors for poorer survival outcomes. Therefore, based on our results, it might be more appropriate to reclassify single large HCC as BCLC stage B instead of the conventional BCLC stage A.

Our study contributes to the ongoing debate regarding the classification of single large HCC within the BCLC staging system. Since its introduction in 1999, the exact position of single large HCC (>5 cm) in the BCLC classification has been a matter of contention among researchers and clinicians.^[4]^ The system has undergone updates over the years, such as the introduction of stage 0, which is a very early stage for small-sized HCC in 2003.^[9,10]^ However, the classification of single large HCC has remained unchanged, leading to inconsistencies in treatment recommendations and confusion in clinical practice.^[2,4,11,12]^

Considering our findings, it is important to reevaluate the position of Group X within the BCLC staging system. Although Group X is classified as early HCC along with Group A, our results showed no significant difference in survival outcomes compared with those of Group B, which had intermediate stage HCC (Fig. 2). Moreover, Group X was identified as an independent risk factor for worse overall survival in multivariate analysis (Table 3). This finding suggests that the current classification of single large HCC as early-stage HCC may not accurately reflect the prognosis of patients.^[13]^

The existing literature on the validity of the BCLC staging system for single large HCC is in agreement with our findings, suggesting that it is reasonable to classify single large HCC as BCLC stage B.^[5,6]^ Wan et al^[5]^ subclassified BCLC stage A as stage A1 (corresponding to Group A in our study) and stage A2 (corresponding to Group X in our study). They found that the overall survival rate of stage A2 was significantly worse than that of stage A1 but comparable to stage B. Cho et al^[6]^ also analyzed the BCLC staging system for single large HCC; in their subgroup analysis, the overall survival of patients with single large HCC (>5 cm) was significantly worse compared with that of patients with smaller HCC. However, their study had its own limitations. They did not perform a survival analysis between the 3 groups as we did in our study. On the other hand, Wan et al (2019) conducted a survival analysis between the 3 groups but did not use propensity score matching, which we employed in our study to minimize the effect of selection bias.

In our multivariate analysis, age over 60 years, PT-INR over 1.2, and creatinine level over 1.2 mg/dL were identified as independent risk factors for worse overall survival (Table 3). The results are consistent with those of previous studies that have highlighted the importance of liver function, as measured by PT-INR^[14–17]^ and creatinine level,^[18–21]^ in predicting survival after curative treatment for HCC.

While interpreting the results of our study, it is essential to acknowledge its limitations and strengths. One of the main strengths of our study is the use of a national cancer database, which increases the generalizability of our findings. Additionally, we employed propensity score matching to minimize the effects of selection bias and confounding factors, leading to well-balanced patient groups for comparison.

Despite its strengths, our study had some limitations. First, it was a retrospective cohort study, which inherently carries the risk of selection bias and unmeasured confounding factors. Second, our patient population predominantly comprised individuals with HBV-related HCC, which is more prevalent in Korea; this may limit the generalizability of our findings to populations with different etiologies of HCC. Finally, it is important to acknowledge that variations in clinical practice may lead to non-adherence to established guidelines for the treatment of liver cancer, as observed in our study. Therefore, we adjusted our treatment methods to reflect real-world scenarios. However, these adjustments may introduce potential limitations that could have influenced the observed survival outcomes. These limitations should be considered when interpreting the results of our study.

In conclusion, compared with smaller HCC, single large HCC (≥5 cm) may represent a distinct group of patients with a worse prognosis. Our findings suggest that these patients should be relocated to BCLC stage B rather than stage A. However, further research is needed to develop tailored treatment approaches for this group of patients, taking into account factors such as tumor biology, liver function, and patient comorbidities.

## Acknowledgments

The authors thank the Korea Central Cancer Registry and Korean Liver Cancer Association.

This work received non-financial support from the Research Supporting Program of the Korean Liver Cancer Association based on data from the Primary Liver Cancer Registry, a project supported jointly by the Korean Liver Cancer Association and Korea Central Cancer Registry, Ministry of Health and Welfare, Korea.

## Author contributions

**Conceptualization:** Hyo-Sin Kim.

**Data curation:** Hyo-Sin Kim, Ho Kyun Lee.

**Formal analysis:** Hyo-Sin Kim.

**Funding acquisition:** Hyo-Sin Kim.

**Investigation:** Hyo-Sin Kim, Soo Jin Na Choi, Ho Kyun Lee.

**Methodology:** Hyo-Sin Kim, Soo Jin Na Choi, Ho Kyun Lee.

**Validation:** Hyo-Sin Kim.

**Writing – original draft:** Hyo-Sin Kim, Soo Jin Na Choi, Ho Kyun Lee.

**Writing – review & editing:** Hyo-Sin Kim, Soo Jin Na Choi, Ho Kyun Lee.
